# Safety of stroboscopic visual training in athletes: a mini-review and proposed framework for adverse-event reporting

**DOI:** 10.3389/fphys.2026.1924867

**Published:** 2026-10-09

**Authors:** Jianjun Li, Youlin Xiao, Shiwei Xiao

**Affiliations:** 1 Xianning Vocational Technical College, Xianning, China; 2 Sports College, Dalian University, Dalian, China; 3 Hubei Preschool Teachers College, Ezhou, China

**Keywords:** athlete safety, harms reporting, perceptual training, sports vision, stroboscopic visual training

## Abstract

Stroboscopic visual training (SVT) uses intermittently occluding eyewear to overload perceptual–cognitive processing, and it has been studied across a growing range of sporting contexts. Its efficacy on visuomotor and cognitive outcomes has now been synthesized by several systematic reviews and meta-analyses; its safety, by contrast, has received little dedicated scrutiny. Drawing on 27 studies of stroboscopic training in athletes identified from PubMed, Scopus, and Web of Science, this mini-review examines how participant safety has been screened, monitored, and reported, and outlines the pathways through which the intervention could plausibly cause harm: visual–vestibular conflict and disorientation during dynamic tasks, transient injury risk arising from deliberately degraded vision, cognitive and mental fatigue, and the largely uncharacterized question of photosensitivity-related risk. We find that structured safety monitoring remains uncommon rather than entirely absent. Three studies clearly reported proactive symptom or adverse-event monitoring, while one additional study described safety oversight without structured proactive symptom surveillance. Only one study reported qualitative tolerability symptoms. The sparse and heterogeneous safety reporting therefore does not allow the safety profile of SVT to be characterized reliably. We propose a minimum screening and adverse-event-reporting framework for SVT, aligned with established harms-reporting standards. Addressing this blind spot is important for wider and safer implementation of SVT across populations and settings.

## Introduction

Vision is the dominant sensory channel for the perception–action coupling that underpins sport (Appelbaum and Erickson, 2018). Stroboscopic visual training (SVT) exploits the observation that performers can extract useful information from intermittent visual samples: liquid-crystal eyewear alternates between transparent and opaque states, intermittently withholding vision so that athletes must perceive, anticipate, and act under degraded input (Appelbaum et al., 2011; Wilkins et al., 2018). This perceptual overload is thought to drive neural adaptation that, on return to normal vision, manifests as faster reaction, more efficient information processing, and improved sport-specific performance (Hülsdünker et al., 2021). Sport-specific applications illustrate the practical relevance of SVT. A 10-week intervention incorporating SVT into badminton-specific drills improved laboratory visuomotor reaction speed in elite youth badminton players (Hülsdünker et al., 2021). Similarly, in youth volleyball players, SVT embedded in sport-specific training produced greater short-term improvements in complex reaction speed and reactive agility than regular training (Zwierko et al., 2023). Because SVT can be layered onto real, sport-specific drills, it carries high ecological validity and has been investigated across a widening range of disciplines.

Reflecting this momentum, the efficacy of SVT in athletes has, within roughly the past year, been synthesized by multiple systematic reviews and meta-analyses (Ren et al., 2025; Jothi et al., 2025b). These syntheses converge on modest but reasonably consistent benefits for reaction speed, processing speed, and selected sport-specific skills. Yet a conspicuous asymmetry has emerged: while the potential benefits of SVT are increasingly quantified, its potential costs—discomfort, adverse events, and safety risks—remain almost entirely unexamined. Indeed, despite these recent syntheses, no review has specifically evaluated the safety of SVT.

This gap matters. SVT is unusual among training methods in that its active ingredient is the deliberate degradation of a primary sensory channel, frequently while athletes perform fast, dynamic, whole-body movements. The same manipulation that is intended to be beneficial is also, on its face, one that could provoke disorientation, falls, or symptoms in susceptible individuals. Stroboscopic eyewear is commercially available and has attracted interest from coaching and sport-performance practitioners (Carroll et al., 2021). An evidence base for efficacy that is not matched by an evidence base for safety is incomplete.

Accordingly, this mini-review (i) outlines the mechanistic basis for plausible harms from SVT, (ii) critically appraises how safety has been screened, monitored, and reported in studies of SVT in athletes, and (iii) proposes a minimum screening and adverse-event-reporting framework intended to make future SVT research and practice safer and more transparent.

## Why stroboscopic training could cause harm: mechanistic considerations

Three broad pathways make harm from SVT biologically plausible. None has been adequately studied, but each is supported either by indirect evidence or by the precautions that SVT researchers themselves have adopted; the three pathways and the safety-related outcomes to which they plausibly lead are summarized in **Figure 1**.

**Figure 1:**
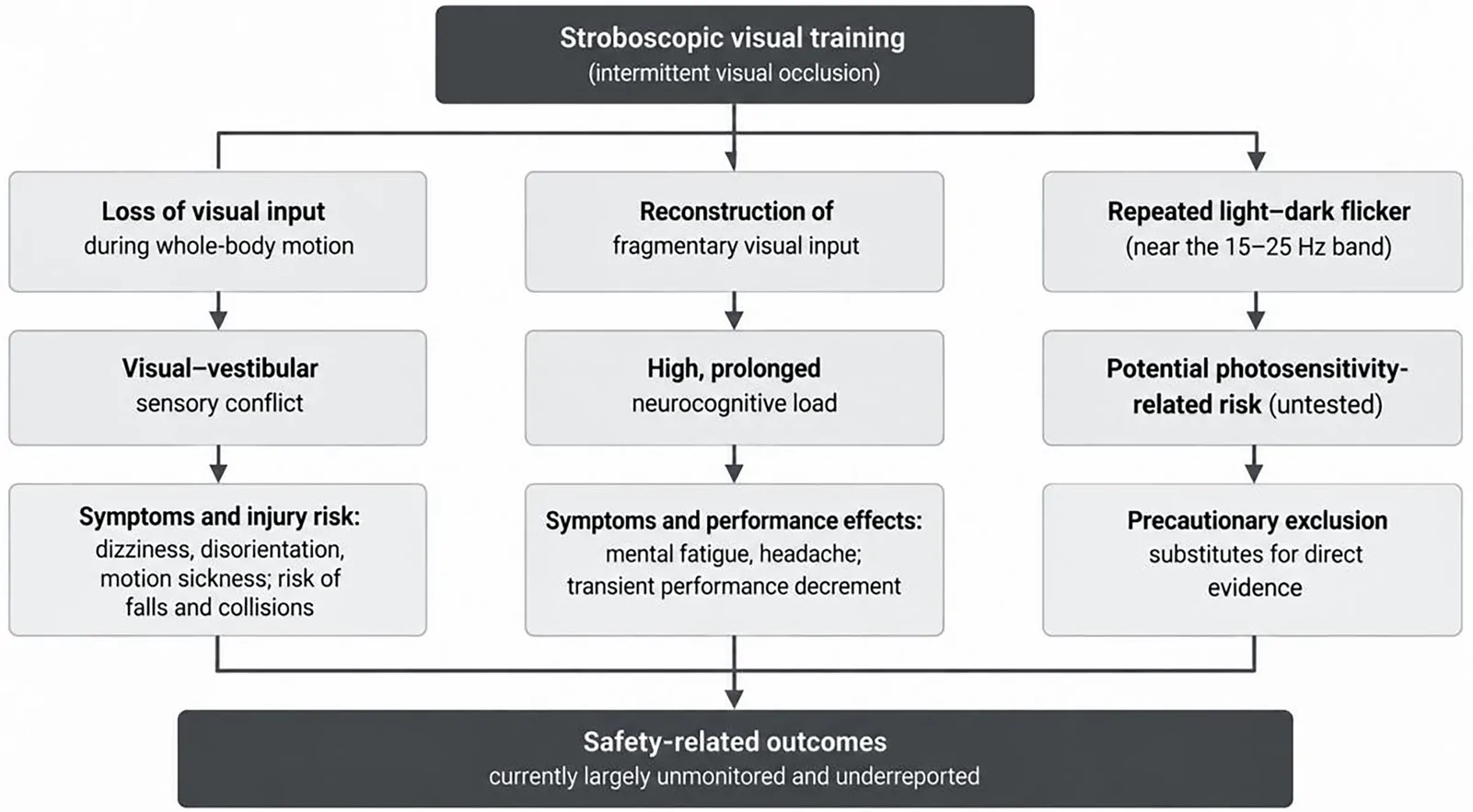
Mechanistic pathways through which stroboscopic visual training could plausibly cause harm. Three non-exclusive pathways—visual–vestibular conflict, neurocognitive load, and a potential but uncharacterized photosensitivity-related risk—converge on safety-related outcomes that current studies largely neither monitor nor report.

## Visual–vestibular conflict, disorientation, and acute injury risk

Stable posture and gaze depend on the integration of visual, vestibular, and proprioceptive signals. Intermittent visual occlusion reduces the temporal availability of visual input but does not necessarily create visual–vestibular conflict. Experimental single-leg balance findings demonstrate impaired postural control under stroboscopic viewing, consistent with reduced visual feedback and altered sensory weighting (Kim et al., 2017); they do not establish sensory conflict as a cause of symptoms. During head or body motion, vestibular and proprioceptive signals remain available through opaque intervals, whereas new visual motion information becomes available only during transparent intervals. A mismatch could arise if intermittently sampled visual motion cues are inconsistent with nonvisual estimates of self-motion, rather than simply less available. However, this mechanism has not been directly demonstrated during athletic SVT. Dizziness, disorientation, and motion-sickness-like symptoms attributable to such conflict therefore remain hypothesized risks, rather than established consequences of SVT. Indeed, 4-Hz liquid-crystal shutter glasses reduced motion sickness during head movements with left–right reversing prisms in a laboratory study (Reschke et al., 2006), indicating that the effects of intermittent vision depend on the viewing and movement conditions. Hülsdünker et al. (2021) restricted training to tasks without head rotation and cautioned that mismatched vestibulo-ocular information might induce discomfort; this was a precaution, not direct evidence that SVT caused sensory conflict or related symptoms. When SVT is layered onto high-speed, dynamic tasks such as skating, jumping, or climbing, the intermittent loss of vision also introduces a plausible acute risk of mis-stepping, collision, or falling.

## Cognitive and mental fatigue

SVT is repeatedly characterized as a high-load neurocognitive stimulus (Vasile and Stănescu, 2023). Sustained effort to reconstruct a coherent percept from fragmentary visual samples imposes substantial cognitive demand, which may accumulate as mental fatigue. This is relevant for two reasons. First, mental fatigue is itself associated with impaired endurance and skilled performance in athletes (Van Cutsem et al., 2017), so an SVT session that leaves an athlete mentally fatigued could transiently degrade—rather than enhance—the very performance it is intended to improve, and could elevate injury risk during subsequent practice. Second, fatigue, dizziness, visual discomfort, and related experiences are tolerability symptoms that can remain undetected unless they are actively solicited. Vasile and Stănescu (2023) identified mental fatigue as a self-reported disadvantage of stroboscopic training in climbers through qualitative post-session feedback.

## Photosensitivity: an assumed but uncharacterized risk

The reviewed studies used several distinct safety-related terms, including a history of seizures, diagnosed epilepsy, migraine, general light sensitivity, marked photosensitivity, and neurological disorders. These terms were retained as reported in the primary studies and were not treated as interchangeable. For the purposes of this review, “photic susceptibility” refers broadly to a potential individual sensitivity to intermittent light–dark stimulation; it is used descriptively rather than diagnostically and is not intended to be synonymous with seizure history, epilepsy, photosensitive epilepsy, migraine, general light sensitivity, or clinically recognized photosensitivity. Appelbaum et al. (2011, 2012) advised individuals with a history of seizures, migraines, or light sensitivity not to participate, whereas other studies applied formal exclusion criteria involving epilepsy, migraine, photosensitivity, or neurological conditions (Ellison et al., 2020; Hülsdünker et al., 2021; Guo et al., 2024; Jothi et al., 2025a; Weng et al., 2026; Xiao et al., 2026; Gultekinler et al., 2026). These precautions reveal a tacit assumption that stroboscopic eyewear carries photic risk. The assumption is understandable: intermittent photic stimulation at flicker frequencies of approximately 15–25 Hz is the most provocative range for photoparoxysmal responses (Fisher et al., 2005; Kasteleijn-Nolst Trenité et al., 2012), and SVT protocols commonly operate at or pass through this band (for example, schedules beginning at 15 Hz; Hülsdünker et al., 2021; Zwierko et al., 2023). However, the provocative stimulus in classical photosensitivity is a high-luminance light flash or a high-contrast pattern, whereas stroboscopic eyewear intermittently darkens the visual field rather than emitting bright flashes. It should be emphasized that occlusion-based stroboscopy nonetheless generates repeated light–dark transitions and therefore constitutes a form of luminance flicker rather than an absence of photic stimulation; the salient difference lies in the luminance profile—a periodic reduction rather than a high-intensity increase in luminance—rather than in the presence or absence of flicker per se. The photic risk of occlusion-based stroboscopy may therefore be considerably lower than that of classical photic stimulation, but it has been neither characterized nor formally refuted. At present, precautionary exclusion substitutes for evidence: studies remove the individuals who might be at risk without establishing whether a risk exists, and frequently without reporting how screening was performed or whether anyone was excluded.

## The current state of safety reporting in athlete SVT studies

Search strategy and study selection. The literature search covered PubMed, Scopus, and Web of Science from database inception to September 2026 using intervention terms (“stroboscopic” OR “strobe” OR “intermittent visual occlusion” OR “Senaptec” OR “Nike Vapor Strobe”) combined with population terms (“athlete” OR “sport” OR “player”). Studies were eligible if they were peer-reviewed, with an English-language full-text version available, applied stroboscopic eyewear or a comparable stroboscopic visual perturbation in athletes, either as an acute or single-session exposure or as a repeated training intervention, and reported visual, motor, performance, or safety-relevant outcomes. A total of 27 eligible studies of stroboscopic training in athlete-defined populations were included in the synthesis. Because this review was designed as a focused mini-review rather than a systematic review, no formal risk-of-bias appraisal or meta-analysis was undertaken. Retrieval counts, duplicate removal, screening totals, and reasons for full-text exclusion are provided in **Figure 2**.

**Figure 2:**
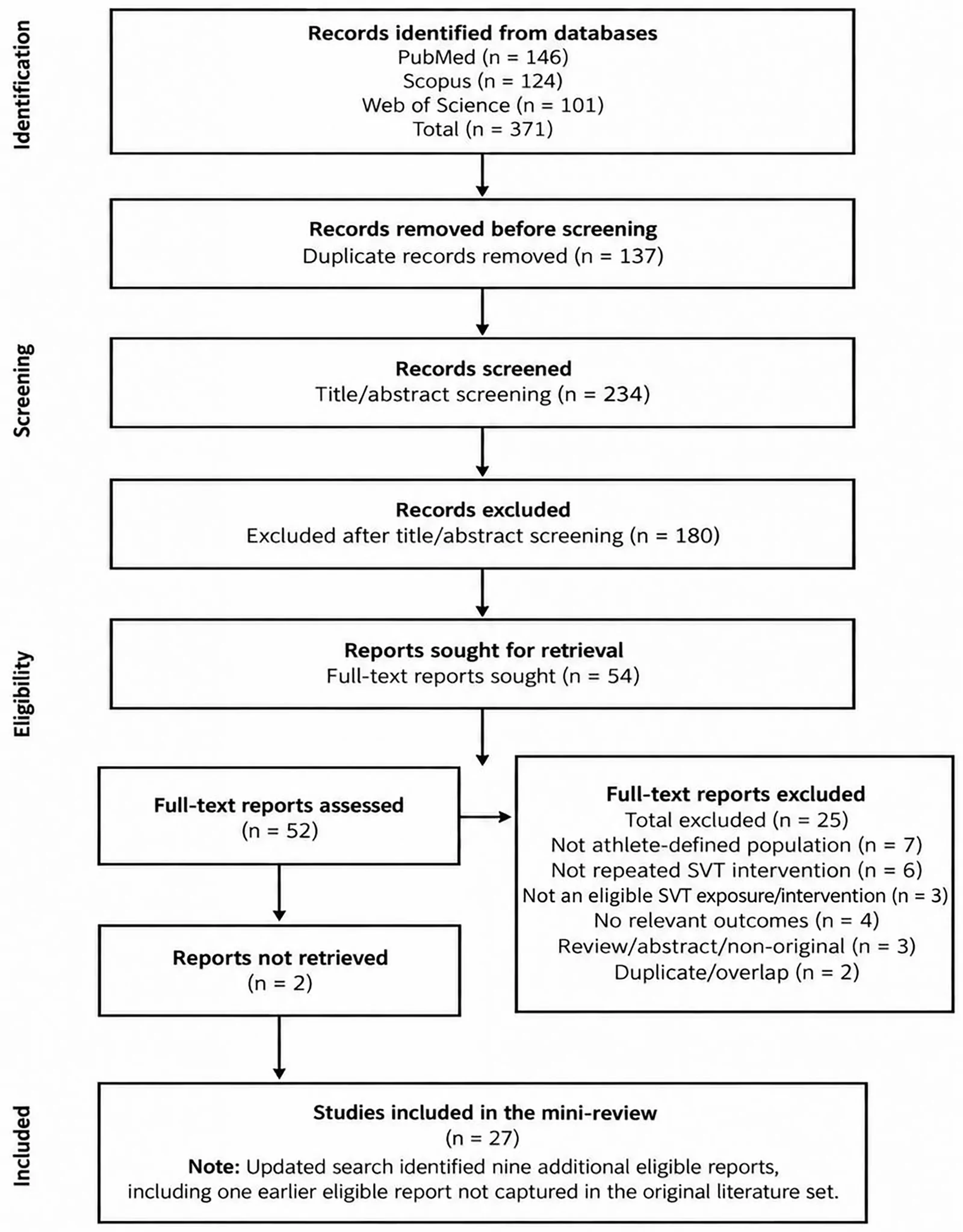
PRISMA-style flow diagram of study identification and selection.

Study selection and data extraction. YX conducted the initial title/abstract and full-text screening. JL independently verified all included studies and full-text exclusions, with disagreements resolved through discussion with XS. JL extracted the study data, and YX independently verified the safety-related variables against the original reports for studies without author overlap. Because YX was a co-author of Xiao et al. (2026), the safety-related extraction for that study was independently verified by XS. The verified variables included population, strobe setting, screening criteria, safety monitoring, reported symptoms or events, adverse-event definitions, and severity grading.

Terminology. For this review, symptoms refer to subjective experiences such as dizziness, headache, visual discomfort, or mental fatigue; hazards refer to conditions that may increase the likelihood of harm, such as degraded vision during dynamic movement; injuries refer to physical events such as falls or collisions; and serious adverse events refer to medically significant events such as seizures requiring clinical attention. These categories were not treated as interchangeable.

Three patterns emerge from the safety-relevant features of these studies (**Table 1**).

**Table 1:** Safety-relevant features and safety-event reporting across the 27 included stroboscopic visual training studies in athletes. AE, adverse event.

Study	Sport / population	Strobe setting	Screening criteria reported	Proactive AE monitoring	AE definition / severity grading	Safety-related symptoms / events reported
Appelbaum et al., 2011	In-lab / ultimate frisbee / football	6→3 Hz	History of seizures, migraines, or light sensitivity (advised not to participate)	Not reported	No / No	Monitoring not reported
Appelbaum et al., 2012	Soccer / basketball	6→3 Hz	History of seizures, migraines, or light sensitivity (advised not to participate)	Not reported	No / No	Monitoring not reported
Mitroff et al., 2013	Ice hockey	8 levels (100 ms clear; 67–900 ms opaque)	Not reported	Not reported	No / No	Monitoring not reported
Wilkins and Gray, 2015	Soccer / basketball (collegiate)	100 ms clear; 25–900 ms opaque	Not specified	Not reported	No / No	Monitoring not reported
Schootemeijer and Visch, 2017	Tennis (U16)	7→5 Hz	Not specified	Not reported	No / No	Monitoring not reported
Wilkins et al., 2018	Soccer goalkeepers	≈6→1 Hz	Not specified	Not reported	No / No	Monitoring not reported
Beavan et al., 2021	Soccer	1 Hz	Not specified	Not reported	No / No	Monitoring not reported
Ellison et al., 2020	Combined sports	4 Hz	Epilepsy excluded; adaptive protocol for undiagnosed photosensitivity (outcome not reported)	Not reported	No / No	Monitoring not reported
Hülsdünker et al., 2021	Badminton	15→9 Hz	Epilepsy, migraine, neurological disorders excluded; head-rotation tasks avoided	Not reported	No / No	Monitoring not reported
Uzlaşır et al., 2021	Athletes with chronic ankle instability	≈4 Hz	Ankle fracture; lower-extremity surgery; head injury <3 mo; chronic musculoskeletal balance condition; neurological problems affecting vision/EEG	Not reported	No / No	Monitoring not reported
Zwierko et al., 2023	Volleyball (youth)	15→9 Hz	Epilepsy, migraine, or injury preventing test completion	Not reported	No / No	Monitoring not reported
Jendrusch et al., 2023	Tennis (n=22; high-/lower-level)	Nike Vapor Strobe; 5.3 / 2.3 / 1.0 Hz; dark phase 88 / 341 / 909 ms	Not specified	Not reported	No / No	Monitoring not reported
Vasile and Stănescu, 2023	Rock climbing	Levels 1–2	Not specified	Yes: post-session feedback questionnaire	No / No	Mental fatigue; dizziness; difficulty distinguishing hold colors; isolated eye tiredness/head discomfort in narrative responses
Guo et al., 2024	Skeet shooting	Not reported	Migraine, epilepsy, neurological disorders, medication use screened	Not reported	No / No	Monitoring not reported
Li et al., 2024	Curling	6→3 Hz	Neurological / musculoskeletal disorders excluded	Not reported	No / No	Monitoring not reported
Zwierko et al., 2024	Handball	5→9 Hz	Not specified	Not reported	No / No	Monitoring not reported
Choi et al., 2024	National athletes with chronic ankle instability (n=40)	Senaptec; 100 ms clear / 150 ms opaque; Level 3 ≈4 Hz	History of fracture or surgery; visual or vestibular impairments excluded	Not reported	No / No	Monitoring not reported
Dendy et al., 2025	Varsity softball (female, 16 yr; case study)	Senaptec; progressive levels 1–8 (hitting levels 1–5); exact duty cycle/frequency not reported	Cleared by Doctor of Optometry; no specific exclusion criteria reported	Not reported	No / No	Monitoring not reported
Jothi et al., 2025a	Soccer	Levels 1→8	Ocular; neurological (including epilepsy/migraine); psychiatric; systemic illness excluded	Not reported	No / No	Monitoring not reported
Fortes et al., 2025	Male national-level soccer (n=28; 14/14)	Nike Vapor Strobe; 100 ms clear / 210 ms opaque; Level 4 ≈4.76 Hz	Normal/corrected vision; no participant excluded	Not reported	No / No	Monitoring not reported
Lu et al., 2025	Soccer (college-aged male)	3 Hz; SVT + balance training	Concussion, vestibular disorder, ankle/lower-extremity injury <3 mo excluded	Not reported	No / No	Monitoring not reported
Li et al., 2026	Basketball (collegiate male, Tier 2)	15→9 Hz; duty cycle 50→60–70%; Nike Vapor Strobe	Concussion; vestibular dysfunction; lower-limb injury <3 mo; clinical condition affecting balance; concurrent visual training excluded	Yes: dizziness, visual fatigue, and nausea monitored/documented throughout intervention	No / No	Monitored; no dizziness, visual fatigue, or nausea observed/reported
Diao et al., 2026	Soccer (college-aged female)	3 Hz (100 ms clear; 233 ms opaque); Senaptec Strobe	Concussion, vestibular disorder, or lower-limb injury <3 mo excluded	Not reported	No / No	Monitoring not reported
Weng et al., 2026	Soccer (male collegiate, National Level 1)	Senaptec Strobe; 15→9 Hz; duty cycle 50→70% over 6 wk	Goalkeepers; epilepsy, seizures, migraine, marked photosensitivity, severe visual impairment, neurological disorder, vestibular dysfunction, and lower-limb injury excluded	Yes: dizziness, visual discomfort, nausea, excessive fatigue, and lower-limb pain monitored each session; training paused if discomfort	No / No	Monitored; no fatigue- or discomfort-related event requiring termination, withdrawal, or medical referral
Xiao et al., 2026	Boxing (female amateur, Olympic-style)	Senaptec Strobe; 10→5 Hz; duty cycle 70→50%	Corrective lenses, pregnancy, chronic neurological conditions (epilepsy, migraine with aura) excluded	Safety oversight reported (sports-medicine physician available); proactive structured symptom surveillance not described	No / No	No adverse events reported
Wang et al., 2026	Badminton (male collegiate; n=20)	EYESTROBE/Senaptec; 3 Hz; 100 ms clear / 230 ms opaque	No lower-limb injury <3 mo; no acute/chronic condition affecting balance/performance; visual/ocular contraindications screened	Not reported	No / No	Monitoring not reported
Gultekinler et al., 2026	Basketball (male, 14–18 yr; n=40)	Senaptec; 100 ms clear / 150 ms opaque	Upper/lower-extremity surgery <1 yr; MSK injury <1 mo; neurological disorders, epilepsy, and ADHD excluded	Not reported	No / No	Monitoring not reported
Summary (n = 27)			Seizure/epilepsy 10; migraine 8; neurological 8; photosensitivity/light sensitivity 4; no specific screening criteria stated 10	3/27 proactive; +1 safety oversight; monitoring not reported 23/27	0/27 with predefined AE definition/severity grading	Symptoms 1/27; monitored/oversight without reportable AE requiring action 3/27; monitoring not reported 23/27

“Not reported” indicates that the full text was reviewed but the relevant safety information was not described.

First, pre-participation screening is inconsistent and rarely justified. Several studies specified neurologically oriented exclusion criteria involving epilepsy, migraine, or neurological disorders (Ellison et al., 2020; Hülsdünker et al., 2021; Guo et al., 2024; Li et al., 2024), whereas Appelbaum et al. (2011, 2012) advised individuals with a history of seizures, migraines, or light sensitivity not to participate. Ten studies did not report specific safety-screening criteria. Where screening was applied, the rationale and procedure were seldom described, making it impossible to judge whether susceptible individuals were reliably identified.

Second, risk-mitigation measures were occasionally adopted but their implementation and outcomes were not reported. Ellison et al. (2020) described an adaptive protocol intended to minimize the risk of seizures arising from undiagnosed photosensitivity, but did not report whether any participant was excluded or experienced symptoms, leaving the protocol’s effectiveness unknown. Hülsdünker et al. (2021) avoided head-rotation tasks on safety grounds, but did not describe systematic adverse-event monitoring, leaving the empirical basis for the precaution unclear. Such measures suggest that researchers perceive potential risks, even as the data needed to evaluate those risks go uncollected.

Third, structured adverse-event monitoring remained uncommon rather than entirely absent. Vasile and Stănescu (2023) provided the clearest earlier example of active participant feedback. In this qualitative study, nine advanced climbers aged 13–19 years provided subjective feedback after each of six training sessions. The assessment was described as a feedback questionnaire and included questions concerning adaptation to the stroboscopic glasses, fatigue, perceived skill development, perceived benefits, and disadvantages experienced during training. Responses were analyzed qualitatively for recurring themes. The principal safety-related disadvantages summarized by the authors were mental fatigue, dizziness, and difficulty distinguishing the colors of some climbing holds; narrative responses also included isolated descriptions of eye tiredness and head discomfort. However, the study did not use a standardized adverse-event checklist, predefined adverse-event definition, symptom-severity scale, or systematic recording of symptom duration. These observations should therefore be interpreted as qualitative self-reported symptoms rather than formally adjudicated adverse events, and they do not establish incidence, severity, duration, or causality.

More recent intervention studies have begun to report safety monitoring more explicitly. Li et al. (2026) studied 42 male collegiate basketball players during an 8-week intervention with three approximately 40-minute sessions per week. Session attendance was recorded, and the investigators stated that dizziness, visual fatigue, and nausea were monitored and documented throughout the intervention; the training sessions were supervised and strobe parameters could be adjusted within pre-specified ranges to preserve tolerability and movement quality. The authors reported that none of these monitored symptoms was observed. However, the study did not report a standardized adverse-event definition, symptom-severity scale, or adverse-event-specific follow-up, so the absence of reported symptoms should be interpreted as evidence from a supervised monitoring procedure rather than as a formal estimate of adverse-event incidence.

Weng et al. (2026) used a more explicitly described session-level safety procedure in 36 male collegiate soccer players completing six weeks of soccer-specific SVT. Participants were screened for conditions considered relevant to risk during stroboscopic exposure, including epilepsy, migraine, neurological disorders, severe visual impairment, vestibular dysfunction, and lower-limb injury. During every training session, research staff and coaches monitored dizziness, visual discomfort, nausea, excessive fatigue, lower-limb pain, and other adverse symptoms, and training was paused if discomfort occurred. Safety ascertainment relied on direct observation and participant self-report. No fatigue- or discomfort-related event requiring training termination, study withdrawal, or medical referral was recorded. Nevertheless, the study did not use a standardized adverse-event, perceived-exertion, or visual-fatigue scale, again limiting comparison of incidence and severity with other SVT studies.

Xiao et al. (2026) reported a different form of safety oversight in 26 female amateur boxers who completed a six-week SVT program. Participants were informed before enrollment about potential risks, including mild eye strain from stroboscopic stimulation, and a sports-medicine physician was available throughout the study to address any adverse reaction. The authors reported that no adverse events occurred during the intervention. This represents clinically relevant safety oversight, but the article did not describe structured proactive symptom questioning or a standardized adverse-event checklist.

Taken together, these studies suggest a gradual shift toward more explicit safety monitoring in the SVT literature, although the methods used to ascertain and report safety outcomes remain heterogeneous. The reported qualitative tolerability symptoms and the absence of monitored symptoms or events in other studies do not permit estimation of adverse-event incidence or characterization of an SVT safety profile. The principal limitation is therefore not simply the small number of reported events, but the sparse, heterogeneous, and insufficiently standardized ascertainment of harms across studies. In this context, the absence of reported adverse events should not be interpreted as evidence of their absence (Altman and Bland, 1995).

Quantitatively, the reporting gap remains substantial (**Table 1**). Strict proactive symptom or adverse-event monitoring was clearly described in 3 of 27 studies. One additional study described safety oversight without structured proactive symptom surveillance. Only one study reported qualitative tolerability symptoms. No predefined adverse-event definition or standardized severity-grading system was identified across the 27 included studies. Accordingly, the updated evidence indicates that systematic harms ascertainment is sparse and methodologically inconsistent, not that SVT has been demonstrated to be free of adverse effects.

This pattern contrasts sharply with norms in clinical and exercise-science trials, where structured harms reporting is expected and supported by current guidance such as CONSORT Harms 2022 (Junqueira et al., 2023). SVT research has not yet adopted comparable practice. The consequence is that, despite a rapidly maturing efficacy literature, the field cannot currently answer basic safety questions: What proportion of athletes experience symptoms? How severe and how durable are they? Do they vary with strobe frequency, duty cycle, cumulative exposure, task, or population? Until adverse events are systematically measured and reported, these questions remain unanswerable.

## Discussion

The limitations of the current evidence are also reflected in the precautions—advice against participation for individuals with seizure, migraine, or light-sensitivity histories (Appelbaum et al., 2011, 2012), formal epilepsy and migraine exclusions (Hülsdünker et al., 2021), adaptive familiarization (Ellison et al., 2020), and avoidance of head rotation (Hülsdünker et al., 2021)—that several groups adopted without consistently reporting their outcomes.A field that simultaneously takes precautions against harm and measures it inconsistently cannot characterize the safety profile of its intervention. The concern is compounded by progressively more demanding parameters and longer cumulative exposures (Wilkins et al., 2018; Hülsdünker et al., 2021) and by more dynamic, sport-specific tasks such as on-ice skating, curling delivery, and lead climbing (Mitroff et al., 2013; Li et al., 2024; Vasile and Stănescu, 2023)—conditions in which mental fatigue may impair performance (Van Cutsem et al., 2017), while visual–vestibular conflict and associated injury risk during SVT remain hypotheses requiring direct investigation and remain inconsistently monitored. Importantly, closing this gap does not require elaborate methodology; proactive symptom surveillance, predefined adverse-event definitions, severity grading, and transparent reporting—routine in clinical and exercise-science trials (Junqueira et al., 2023)—would suffice to convert the present heterogeneous evidence into more usable safety data.

## A proposed minimum safety framework for SVT research and practice

The deficiencies identified above are readily remediable. We propose a minimum framework for safety screening, monitoring, and reporting in SVT, applicable to both research and supervised practice. It is deliberately lightweight, so that it can be adopted without specialized equipment, and it is organized around four components.

First, pre-participation screening. Before exposure, document a personal and family history of seizures; diagnosed epilepsy (including photosensitive epilepsy where applicable); migraine; general light sensitivity or clinically recognized photosensitivity; vestibular or balance disorders; recent concussion or mild traumatic brain injury; and uncorrected visual disorders. These categories should be recorded separately rather than treated as interchangeable. Inclusion and exclusion criteria should be pre-specified and justified, and the number of individuals excluded for safety reasons should be recorded.

Second, graded exposure and supervision. Begin with brief, low-difficulty familiarization and progress gradually. Provide one-to-one supervision during dynamic or high-speed tasks, ensure adequate space and safe surfaces to mitigate fall and collision risk, and defer high-risk movements (such as unsupported head rotation) until tolerance is established.

Third, structured adverse-event monitoring. Proactively solicit symptoms after every session using a brief, standardized checklist that includes dizziness, headache, nausea or motion sickness, visual discomfort or afterimages, disorientation, and mental fatigue, recording symptom severity and time course. Record any within-session or between-session dropout attributable to symptoms.

Fourth, transparent reporting. Report the screening criteria and the number excluded, the method used to ascertain adverse events, the incidence and nature of any events, adverse-event-related dropouts, and the full exposure parameters—transparent and opaque durations in milliseconds, frequency, duty cycle, and cumulative exposure. Reporting should be aligned with current harms-reporting guidance (Junqueira et al., 2023).

Adopting this framework would impose minimal burden while transforming the evidence base. Even a handful of studies that actively monitored and transparently reported adverse events would allow the field to estimate the incidence and determinants of harm, to define contraindications, and to establish safe dosing—precisely the information that coaches and clinicians need before wider implementation of SVT.

## Limitations

This review has limitations. It is a focused mini-review rather than a systematic review: the search, though spanning three major databases and updated through September 2026, was not exhaustive and was restricted to peer-reviewed reports for which an English-language full-text version was available, and no formal risk-of-bias appraisal or meta-analysis was undertaken. The updated search identified nine additional eligible reports, including an earlier eligible report that had not been captured in the initial June 2025 literature set. Safety-related information was frequently absent from primary reports, even when full texts were available. The underlying evidence base—heterogeneous, generally small studies that seldom collected safety data—further constrains any synthesis. These constraints reinforce rather than weaken the central message: the safety profile of SVT cannot currently be characterized reliably because the necessary data have not been collected consistently.

## Conclusion and future directions

Safety reporting in athlete SVT studies remains sparse and heterogeneous, preventing reliable characterization of the incidence, severity, and determinants of harm. The low number of reported adverse events does not establish either the safety or unsafety of SVT, and falls, collisions, and seizures remain potential rather than established risks because they were not documented as outcomes in the reviewed studies. Future SVT research should treat safety as a core outcome alongside efficacy by using predefined safety outcomes, proactive adverse-event monitoring, and transparent harms reporting; prospective interventional studies should, where appropriate, prospectively register an explicit safety and adverse-event plan.
