# Supplementary material for: Safety of stroboscopic visual training in athletes: a mini-review and proposed framework for adverse-event reporting
Source: Front Physiol. 2026 Oct 9;17:1924867. doi: 10.3389/fphys.2026.1924867 (PMC13691591; doi:10.3389/fphys.2026.1924867)
Supplement: Supplementary file 1 [file DataSheet1.docx]

**Table 1. Safety-relevant features and safety-event reporting across the 27 included stroboscopic visual training studies in athletes. AE, adverse event.**

| Study | Sport / population | Strobe setting | Screening criteria reported | Proactive AE monitoring | AE definition / severity grading | Safety-related symptoms / events reported |
| --- | --- | --- | --- | --- | --- | --- |
| Appelbaum et al., 2011 | In-lab / ultimate frisbee / football | 6→3 Hz | History of seizures, migraines, or light sensitivity (advised not to participate) | Not reported | No / No | Monitoring not reported |
| Appelbaum et al., 2012 | Soccer / basketball | 6→3 Hz | History of seizures, migraines, or light sensitivity (advised not to participate) | Not reported | No / No | Monitoring not reported |
| Mitroff et al., 2013 | Ice hockey | 8 levels (100 ms clear; 67–900 ms opaque) | Not reported | Not reported | No / No | Monitoring not reported |
| Wilkins & Gray, 2015 | Soccer / basketball (collegiate) | 100 ms clear; 25–900 ms opaque | Not specified | Not reported | No / No | Monitoring not reported |
| Schootemeijer & Visch, 2017 | Tennis (U16) | 7→5 Hz | Not specified | Not reported | No / No | Monitoring not reported |
| Wilkins et al., 2018 | Soccer goalkeepers | ≈6→1 Hz | Not specified | Not reported | No / No | Monitoring not reported |
| Beavan et al., 2021 | Soccer | 1 Hz | Not specified | Not reported | No / No | Monitoring not reported |
| Ellison et al., 2020 | Combined sports | 4 Hz | Epilepsy excluded; adaptive protocol for undiagnosed photosensitivity (outcome not reported) | Not reported | No / No | Monitoring not reported |
| Hülsdünker et al., 2021 | Badminton | 15→9 Hz | Epilepsy, migraine, neurological disorders excluded; head-rotation tasks avoided | Not reported | No / No | Monitoring not reported |
| Uzlaşır et al., 2021 | Athletes with chronic ankle instability | ≈4 Hz | Ankle fracture; lower-extremity surgery; head injury <3 mo; chronic musculoskeletal balance condition; neurological problems affecting vision/EEG | Not reported | No / No | Monitoring not reported |
| Zwierko et al., 2023 | Volleyball (youth) | 15→9 Hz | Epilepsy, migraine, or injury preventing test completion | Not reported | No / No | Monitoring not reported |
| Jendrusch et al., 2023 | Tennis (n=22; high-/lower-level) | Nike Vapor Strobe; 5.3 / 2.3 / 1.0 Hz; dark phase 88 / 341 / 909 ms | Not specified | Not reported | No / No | Monitoring not reported |
| Vasile & Stănescu, 2023 | Rock climbing | Levels 1–2 | Not specified | Yes: post-session feedback questionnaire | No / No | Mental fatigue; dizziness; difficulty distinguishing hold colors; isolated eye tiredness/head discomfort in narrative responses |
| Guo et al., 2024 | Skeet shooting | Not reported | Migraine, epilepsy, neurological disorders, medication use screened | Not reported | No / No | Monitoring not reported |
| Li et al., 2024 | Curling | 6→3 Hz | Neurological / musculoskeletal disorders excluded | Not reported | No / No | Monitoring not reported |
| Zwierko et al., 2024 | Handball | 5→9 Hz | Not specified | Not reported | No / No | Monitoring not reported |
| Choi et al., 2024 | National athletes with chronic ankle instability (n=40) | Senaptec; 100 ms clear / 150 ms opaque; Level 3 ≈4 Hz | History of fracture or surgery; visual or vestibular impairments excluded | Not reported | No / No | Monitoring not reported |
| Dendy et al., 2025 | Varsity softball (female, 16 yr; case study) | Senaptec; progressive levels 1–8 (hitting levels 1–5); exact duty cycle/frequency not reported | Cleared by Doctor of Optometry; no specific exclusion criteria reported | Not reported | No / No | Monitoring not reported |
| Jothi et al., 2025a | Soccer | Levels 1→8 | Ocular; neurological (including epilepsy/migraine); psychiatric; systemic illness excluded | Not reported | No / No | Monitoring not reported |
| Fortes et al., 2025 | Male national-level soccer (n=28; 14/14) | Nike Vapor Strobe; 100 ms clear / 210 ms opaque; Level 4 ≈4.76 Hz | Normal/corrected vision; no participant excluded | Not reported | No / No | Monitoring not reported |
| Lu et al., 2025 | Soccer (college-aged male) | 3 Hz; SVT + balance training | Concussion, vestibular disorder, ankle/lower-extremity injury <3 mo excluded | Not reported | No / No | Monitoring not reported |
| Li et al., 2026 | Basketball (collegiate male, Tier 2) | 15→9 Hz; duty cycle 50→60–70%; Nike Vapor Strobe | Concussion; vestibular dysfunction; lower-limb injury <3 mo; clinical condition affecting balance; concurrent visual training excluded | Yes: dizziness, visual fatigue, and nausea monitored/documented throughout intervention | No / No | Monitored; no dizziness, visual fatigue, or nausea observed/reported |
| Diao et al., 2026 | Soccer (college-aged female) | 3 Hz (100 ms clear; 233 ms opaque); Senaptec Strobe | Concussion, vestibular disorder, or lower-limb injury <3 mo excluded | Not reported | No / No | Monitoring not reported |
| Weng et al., 2026 | Soccer (male collegiate, National Level 1) | Senaptec Strobe; 15→9 Hz; duty cycle 50→70% over 6 wk | Goalkeepers; epilepsy, seizures, migraine, marked photosensitivity, severe visual impairment, neurological disorder, vestibular dysfunction, and lower-limb injury excluded | Yes: dizziness, visual discomfort, nausea, excessive fatigue, and lower-limb pain monitored each session; training paused if discomfort | No / No | Monitored; no fatigue- or discomfort-related event requiring termination, withdrawal, or medical referral |
| Xiao et al., 2026 | Boxing (female amateur, Olympic-style) | Senaptec Strobe; 10→5 Hz; duty cycle 70→50% | Corrective lenses, pregnancy, chronic neurological conditions (epilepsy, migraine with aura) excluded | Safety oversight reported (sports-medicine physician available); proactive structured symptom surveillance not described | No / No | No adverse events reported |
| Wang et al., 2026 | Badminton (male collegiate; n=20) | EYESTROBE/Senaptec; 3 Hz; 100 ms clear / 230 ms opaque | No lower-limb injury <3 mo; no acute/chronic condition affecting balance/performance; visual/ocular contraindications screened | Not reported | No / No | Monitoring not reported |
| Gultekinler et al., 2026 | Basketball (male, 14–18 yr; n=40) | Senaptec; 100 ms clear / 150 ms opaque | Upper/lower-extremity surgery <1 yr; MSK injury <1 mo; neurological disorders, epilepsy, and ADHD excluded | Not reported | No / No | Monitoring not reported |
| Summary (n = 27) |  |  | Seizure/epilepsy 10; migraine 8; neurological 8; photosensitivity/light sensitivity 4; no specific screening criteria stated 10 | 3/27 proactive; +1 safety oversight; monitoring not reported 23/27 | 0/27 with predefined AE definition/severity grading | Symptoms 1/27; monitored/oversight without reportable AE requiring action 3/27; monitoring not reported 23/27 |

Note. “Not reported” indicates that the full text was reviewed but the relevant safety information was not described.

**Figure 1. Mechanistic pathways through which stroboscopic visual training could plausibly cause harm. Three non-exclusive pathways—visual–vestibular conflict, neurocognitive load, and a potential but uncharacterized photosensitivity-related risk—converge on safety-related outcomes that current studies largely neither monitor nor report.**

**Figure 2. PRISMA-style flow diagram of study identification and selection**
